# A Brief Writing Intervention Assists Athletes to Cope With Performance Failures

**DOI:** 10.1002/pchj.70026

**Published:** 2025-06-27

**Authors:** David L. Neumann, Mikayla McInnes

**Affiliations:** ^1^ School of Applied Psychology Griffith University Nathan Queensland Australia; ^2^ Griffith Centre for Mental Health Griffith University Nathan Queensland Australia

**Keywords:** affect, athletes, performance failures, rumination, writing intervention

## Abstract

Significant performance failures by athletes can have detrimental psychological effects, potentially leading to maladaptive coping mechanisms, performance anxiety, and psychological distress. The current study investigated if a brief writing intervention could combat the negative impact of recalled performance failures in a sample of competitive athletes. Initially, the athletes recalled an instance of a significant performance failure. Next, an Intervention group completed an expressive writing task based on the principles of cognitive restructuring and reframing, and a Control group completed a neutral writing task. The results indicated that both groups showed improvements in the outcome measures from pre‐test to post‐test. However, the magnitude of the reduced self‐critical rumination behaviors, increased positive affect, and reduced negative affect was greater in the Intervention group than in the Control group. The current study highlights how an expressive writing task can combat negative rumination thoughts and affective reactions. Such brief interventions could be used by athletes during a break in a game or immediately following a game to address performance failures as they happen.

Despite considerable training and effort, professional athletes can make mistakes that negatively impact their performance and mental state. These performance failures may increase the risk of long‐term psychological harm such as performance anxiety, depression, and substance abuse due to promoting dysfunctional beliefs about oneself (Cowden and Worthington 2019; Li et al. 2021). For these reasons, several interventions have been tailored to combat the negative psychological impact of performance failures. However, these interventions are time intensive, may not be suited to athletic populations, or fail to address the performance failures as soon as possible after they occur (Wang et al. 2023). Accordingly, the current study aimed to examine the nature of performance failures by athletes and whether a brief writing intervention can assist athletes in managing the negative psychological impact of these failures.

Research shows that athletes are at a higher risk of developing a mental health disorder due to peer and public exposure, high expectations, relocation, and pressure to perform well (Åkesdotter et al. 2020; Neumann et al. 2023a, 2023b; Sundgot‐Borgen and Torstveit 2004). In particular, at least 34% of athletes experience mental health issues due to earlier career performance failures (Åkesdotter et al. 2020; Li et al. 2021). A performance failure can increase the chances of making performance failures in the future and lead to long‐term lowered self‐worth and self‐confidence, and increased anxiety and drug use (Hill et al. 2019). For reasons such as these, researchers have explored the psychological mechanisms that are associated with performance failures and approaches that might combat their negative effects.

It has been suggested that athletes are more susceptible to distress after a performance failure because of their tendency to engage in persistent unhelpful coping mechanisms (Rice et al. 2016; Rocha and Osório 2018; Sagar et al. 2007). For example, Sagar et al. (2007) reported that athletes are more likely to engage in behavioral and psychological avoidant behaviors such as cognitive distancing and limiting their participation in training or team activities due to feelings of humiliation after a performance mistake. Similarly, Ceccarelli et al. (2019) found that athletes often engage in severe and perpetual self‐criticizing behaviors following a performance failure. Hill et al. (2019) reported that athletes can experience a lowering of personal performance standards, limited attention/emotional control, and negative affect in the short‐term.

Despite the importance of unhelpful coping mechanisms and altered affect, few studies have targeted these processes immediately following the performance failure. Indeed, most approaches have focused on the anxiety and fear of failure before a performance failure happens (Sagar et al. 2010; Taylor et al. 2021). However, this approach may not be flexible or targeted enough to address the more immediate impacts of a performance failure, such as that experienced during a game situation or immediately following the game. To address this issue, it is necessary to develop intervention approaches that are simple, brief, and can be implemented quickly.

There is extensive research on the effectiveness of using brief interventions to combat instances of performance failures within the general population. Self‐compassion, mindfulness, and cognitive behaviour therapy (CBT) interventions have been used for instances of musical and academic performance failures, among other types of failures (Hoyert and O'Dell 2006; Sparks 2022; Torsney et al. 2021; Zhukov 2019). Self‐compassion has been used to combat performance failures with athletes. However, research by Mosewich et al. (2023) and Sutherland et al. (2014) that used a self‐compassion writing task recognizes that some athletes may be resistant to self‐compassion approaches due to fear of being viewed negatively by others or fear of becoming complacent. Other researchers argue for the benefits of mindfulness in combination with CBT to treat internal self‐criticizing behaviours as these models adopt positive ways to challenge thoughts and maladaptive behaviours (Hoffman and Hanrahan 2012; Kenny 2005; Spahn et al. 2016).

In instances of musical performance and academic performance failures, brief CBT techniques such as reframing and cognitive restructuring of thoughts have had positive effects on performance and feelings of guilt, shame, and worry (Kendrick et al. 1982; King 2001; Nagel et al. 1989; Schwerdtfeger et al. 2021). Reframing a thought can be achieved by changing the perspective and finding a learning opportunity from the thought (David 2013). Cognitive restructuring encourages people to challenge negative thought processes and mental representations by reimagining thoughts in an alternative way (Clark 2013). As derived from Beck's cognitive model (Beck and Haigh 2014), cognitive restructuring techniques such as seeking evidence for and against automatic thoughts, altering negative automatic thinking and behaviors, and using self‐comparison in more positive ways can significantly reduce distress, anxiety, and rumination (Dugas et al. 2003; Kendrick et al. 1982; Roland 1993).

There have been few instances in which reframing and cognitive restructuring has been used for athletes within a sport setting to address performance failures. Some researchers have reported reduced performance anxiety and increased self‐confidence and performance within a game setting, but they used intense cognitive restructuring sessions well after the game was completed (Gustafsson et al. 2017; Klockare et al. 2022; Sagar et al. 2009). Notably, Haney (2004) used cognitive restructuring on a small sample of female athletes who were experiencing anxiety and stress due to their sport more generally. The approach began with identifying negative thoughts followed by drawing upon this perpetuating negative thought. Next, the athletes challenged these negative thoughts through Socratic questioning such as “what would I tell a friend who was also thinking this way?” Finally, the athletes replaced this negative thought with a more positive thought. Similar studies conducted among athletes have shown to be effective in reducing overall anxiety and stress (Crocker et al. 1988; Greenspan and Feltz 1989; Kerr and Leith 1993; Kerwin and Bopp 2014; Motevalli et al. 2022).

The techniques of reframing and cognitive restructuring can be implemented in a controlled and brief way through an expressive writing task. Expressive writing is an approach that asks participants to write about their thoughts and feelings about an event, typically over the course of several days (Robertson et al. 2023). Participants are given instructions which ask them to reflect deeply on their thoughts and feelings and not be concerned about their spelling or grammar. Instructions can be open, asking participants to write in an unstructured way, or the instructions can give guidance on areas to focus on in their writing. The latter approach can allow for the writing intervention to incorporate CBT principles designed to combat anxiety, rumination, and poor performance (DiMenichi et al. 2019; King 2001). The length and number of writing sessions can also be adapted, with even brief interventions proving to be effective (Moffitt et al. 2023). Expressive writing interventions have been used in various non‐clinical contexts including the management of stress at work (Lukenda et al. 2024), anxiety in attending college (Robertson et al. 2021), and anxiety in performing music (Tang and Ryan 2020).

Expressive writing approaches have been examined in athlete populations for the management of injury‐related stress, performance anxiety, and career challenges. Mankad et al. (2009b) used an expressive writing task over a 3‐day intervention period with an injured state‐level athlete. The writing sessions focused on disclosing negative emotions associated with injury and rehabilitation. Following the intervention, the athlete showed reduced stress and mood disturbance and reported improved self‐confidence and self‐esteem. However, the case study design limits generalization and causal inferences from this study. Other studies have recruited samples of athletes from a range of sports who have experienced various psychological problems, such as performance anxiety, injury‐related stressors, and career challenges. These studies report that expressive writing interventions have led to less psychological stress and mood disturbance (Mankad et al. 2009a), lower grief (Mankad and Gordon 2010), improved emotion management, problem‐solving, and self‐development (Hudson and Day 2012), and reduced cognitive anxiety (Jannah et al. 2019) following the intervention when compared to before the intervention. While these results are promising, the designs of these studies did not include a control group. As a result, it is not possible to make definitive conclusions regarding the causal role of the writing intervention on improving athletes' psychological state.

A randomised control trial design was used in a study by Duncan et al. (2013) with a sample of injured athletes. The intervention consisted of the athletes writing about their injury onset, the emotions and impact of their injury, and their future coping and psychological growth. A control group wrote about non‐injury and non‐emotional topics. The intervention group showed significantly improved physical mobility associated with their injury relative to the control group following the intervention. Improvements were also observed in positive and negative emotions and in injury‐related anxiety following the intervention, although these were not significantly different from the control group. Taken together, there is some evidence that writing interventions may be effective in helping athletes manage psychological problems associated with their sport participation. In addition to its psychological benefits, the ease, cost, and feasibility of implementing a writing intervention facilitate its adoption in practice (DiMenichi et al. 2019; Hudson and Day 2012; Ramirez and Beilock 2011). However, the benefits of the approach to relieve self‐critical thoughts, rumination, and altered affect for athletes who have experienced performance failures remain to be demonstrated.

## 1 The Present Study

Although there is evidence that cognitive restructuring interventions may combat instances of performance failures, it remains to be tested in a sport setting using an approach that is designed to be brief and implemented proximal to a performance failure. Therefore, we aimed to test if a brief expressive writing task based on the CBT techniques of cognitive restructuring and reframing thoughts can be used in instances of performance failures for athletes. Performance failures are relatively rare and unpredictable. For this reason, we tested the intervention by asking athletes to recall an instance in which they made a significant performance failure. We used thematic analysis from written responses to explore the nature of the performance failures and their accompanying negative thoughts to inform future intervention approaches. In addition, we tested whether the writing intervention had a beneficial effect on self‐critiquing behavior and affect when measured after the intervention (post‐test) relative to before (pre‐test) and relative to a control condition. Two hypotheses were tested:

*The intervention will have a beneficial effect across groups in that self‐critical rumination and negative affect will be lower and positive affect will be higher in an intervention group than in a control group at post‐test*.

*The intervention will have a beneficial effect across time in that the difference between pre‐test and post‐test scores for self‐critical rumination, negative affect, and positive affect will be greater for an intervention group than for a control group*.

## 2 Method

### 2.1 Participants

Participants were recruited via the first‐year participant pool of introductory psychology courses in exchange for partial course credit, or via an Instagram post, or word of mouth in exchange for the chance to win an AUD$50 gift voucher. Eligible participants were required to be currently playing at a regional or higher level and had experienced a significant performance failure within the last 2 years. A significant performance failure was defined as an influential mistake that occurred during a sport setting, such as a missed goal or tackle or letting the team/self/public down in some way. An a priori sample size was calculated using G*Power (Faul et al. 2007) and the parameters of 0.8 for power, *f* = 0.25 for a medium effect size, and significance of *α* = 0.05. The medium effect size parameter estimate represents the average of the effect sizes calculated from the study by Jannah et al. (2019) for their pre‐ to post‐intervention effect for cognitive state anxiety (*d* = 0.65) and the study by Duncan et al. (2013) for the intervention and control group difference for injury mobility (*d* = 0.36). Cohen (1988) notes that a medium effect represents an effect that is large enough to be detected by a casual observer. The sample size calculation indicated that 34 participants (*n* = 17 per group) were required to detect an interaction for a 2 × 2 mixed factorial ANOVA.

Participants (*N* = 35) were randomly allocated to an Intervention group (*n* = 18; 9 = male, 9 = female) or a Control group (*n* = 17; 10 = male, 7 = female). Random allocation was not constrained to keep group sizes equal but continued until at least 17 participants were in each group. Participants in the Intervention group were aged 18 to 27 years (*M* = 22.83, SD = 2.85) and participants in the Control group were aged 18 to 27 years (*M* = 22.85, SD = 2.83). The sport and competition level played by participants in the Intervention and Control groups are shown in Table 1. As can be seen, the sample consisted of participants who played a variety of sports, with association football (soccer) and touch football being the most represented. The highest level of competition varied, with most at the state or regional level.

**TABLE 1:** Number of participants who played each sport and competition level across the intervention (*n* = 18) and control (*n* = 17) groups.

Sport	Intervention	Control
Soccer	4	4
Touch Football	3	9
Netball	2	0
Muay Thai	0	0
Judo	1	0
Beach Volleyball	0	1
Hockey	1	0
Cheerleading	1	0
Cricket	1	0
Rugby 7's	1	0
Badminton	1	0
AFL	0	1
Figure Skating	1	0
Swimming	1	0
Basketball	1	0
Golf	0	1
Competition level		
International	0	1
National	5	3
State	3	7
Regional	11	5

### 2.2 Measures

#### 2.2.1 Modified Self‐Critical Rumination Scale

The 10‐item adapted Self‐critical rumination scale (SCRS; Smart et al. 2016) was used to measure severity of self‐critical rumination from a performance failure. This scale uses a 4‐point scale (1 = *not at all*, 4 = *very much*). Higher scores indicate higher levels of self‐critical rumination. The authors report good internal consistency with a reliability coefficient of *α* = 0.92 and good test–retest reliability of *α* = 0.86 (Smart et al. 2016). All items were modified from the original scale to be more appropriate to the nature of the participants and testing context. This was done by replacing self‐ or other‐focused phrases (e.g., “myself”, “I'd said or done”, “I have made”) with failure focused phrases (e.g., “my performance failure”). An example item is “I criticize myself a lot for how I acted during my performance failure”. The scale had a Cronbach's value in the present study of *α* = 0.87 at pre‐intervention and *α* = 0.94 at post‐intervention.

#### 2.2.2 Positive and Negative Affect Scale

The 20‐item positive and negative affect scale (PANAS) (Watson et al. 1988) was used to measure emotional state. Ten items measured positive affect and 10 items measured negative affect using a 5‐point scale (1 = *very slightly or not at all*, 5 = *extremely*). Sample items included interested, strong, and enthusiastic for the positive subscale, and upset, guilty, and ashamed for the negative subscale. Higher scores indicate higher levels of affect. The authors report acceptable convergent and discriminant validity and internal consistency reliability coefficients of *α* = 0.90 for the positive affect subscale and *α* = 0.87 for the negative affect subscale (Watson et al. 1988). As assessed by Cronbach's *α*, acceptable internal consistency was found in the present study for the positive subscale at pre‐intervention (*α* = 0.86) and post‐intervention (*α* = 0.95) and for the negative subscale at pre‐intervention (*α* = 0.91) and post‐intervention (*α* = 0.91).

#### 2.2.3 Modified Visual Imagery Questionnaire

The 3‐item adapted Visual Imagery Questionnaire (VIQ; Marks 1973) was used to measure the participants vividness of their recalled performances failure. An example item included “When recalling this performance failure, how vivid is this memory?”. Responses are made on a 5‐point scale (*1 = No image at all, only you ‘know’ that you are thinking of the object* to *5 = Perfectly clear and as vivid as normal vision*). Higher scores indicated higher accuracy and vividness of the recalled performance failure. The VIQ showed acceptable internal consistency in the present study with Cronbach's *α* = 0.70.

### 2.3 Procedure

The current study used a cross‐sectional design with a protocol approved by the Griffith University Human Research Ethics Committee. Participants completed the study in person on the university campus or at their training venue privately in a single session. Initially, participants provided informed consent and provided demographic information. Next, participants were given the following instructions,

Please take 1 min to recall a recent performance failure or error in your sport that led you to engage in negative self‐critiquing behaviour, rumination, and lower mood during a match. For example, you caused a penalty shot for the opposing team, missed a goal or a tackle, and this led you to start thinking negatively about your performance where you judged yourself, or you compared yourself, or were constantly thinking negative and unhelpful thoughts due to this performance failure. Now that you have had time to recall this failure, please write down in as much detail as you can the details of this failure including what happened, how it happened, who witnessed this, what were you thinking at the time, and what emotions and feelings were present at the time and after the performance failure. Please continue writing for 5 min in as much detail as you can.

To control for time spent during the recall, participants were asked to continue writing until 5 min had elapsed. After the recall period, participants completed the VIQ, SCRS, and PANAS.

Next, participants were randomly allocated to either the Intervention group or Control group. For the Intervention group, a brief written cognitive intervention was administered in which they wrote down their responses on paper for 5 min. The responses aimed to minimize and challenge any negative emotions/thoughts that had arisen from recalling a recent performance failure that led to self‐critiquing behaviour and negative emotions/thoughts. Based on the literature, participants were asked to write down answers to questions of “Why do you identify this experience as a performance failure?” and “What is a negative thought that comes to mind when you think about your performance failure?” and to identify a perpetuating negative thought (Automatic Thoughts) (Hope et al. 2010). Participants next wrote responses to the questions of “How could you change this thought into a learning opportunity”, and “Knowing what you know now, what would you do differently in the future?”, to challenge maladaptive thoughts and change them to adaptive thought processes (Thought Reframing) (David 2013).

The Control group completed a neutral writing task where they were instructed to recall a warmup routine that they would normally engage in prior to their sport. They were asked to continue writing in as much detail as they could for 5 min. To prompt the participants, they were asked to “Please describe this routine, including the specific exercises or stretches that you would engage in, and where this warmup routine would usually take place”. In addition, participants were asked to “Write about one thing that you have learnt that has improved the way you warm up in the future”. To control for time spent, participants continued writing until 5 min had elapsed. Following the writing period for both groups, the PANAS and SCRS was readministered to provide a post‐test measure of affect and self‐critical rumination.

### 2.4 Statistical Analysis

The written responses obtained when participants wrote about their performance failure and when completing the brief writing CBT intervention were examined. Participants on average wrote 139 words (about half an A4 page) to describe their performance failure and 84 words when reflecting on their negative thoughts and emotions during the intervention. The analysis used thematic coding to identify major themes in the types of performance failures recalled, the negative thoughts and emotions experienced during the recalled performance failures, and the negative thoughts and emotions reported during the intervention (Intervention group only). Performance failures were defined as a failure within a game setting that led to thinking negatively about oneself or their performance and resulted in engaging in self‐critiquing behaviours. Coding was initially completed by an experimenter. To cross‐check the validity of the coding categories, a second expert in the area was consulted. During this process, some themes were grouped and non‐psychological impacts (e.g., injury) were removed. Some performance failures for different sports followed a similar theme and were coded in the same category. For example, “Letting a Goal Through”, “Letting a Try Through”, “Letting a Punch Through”, and “Letting a Player Through” were coded together as it was defined as something getting past the athlete. Similarly, some themes were grouped together in the coding of negative thoughts. For example, “Overthinking” and “Anxious” were coded together to define constant thoughts, stress, worry, and rumination surrounding the performance failure.

The SCRS scores, PANAS positive scores, and PANAS negative scores were examined with separate 2 × (Group: Control vs. Intervention) × 2 (Time: Pre‐test vs. Post‐test) mixed factorial ANOVAs. H1 as tested by examining the Group × Time interaction and post hoc *t*‐tests to compare the dependent variables between groups separately at pre‐test and post‐test. To examine the magnitude of the change in scores following the intervention, change scores were calculated by subtracting pre‐test scores from post‐test scores. H2 was tested by comparing the change scores between groups with *t*‐tests. In addition, the 95% confidence intervals of the change scores were calculated. Confidence intervals that did not contain zero indicates that change across time was significant. An independent groups *t*‐test was conducted to compare groups in VIQ scores. An α‐level (two‐tailed) of 0.05 was used for all analyses.

Prior to the analysis using SPSS version 28, the data were screened to check for data entry errors and missing values. Upon observation of the normal plots and histograms, the assumption of normality was met as most Shapiro Wilk values did not exceed the cut off value of *p* < 0.01, and most scores followed the trend line. The exception to this was PANAS Positive scores at Pre‐test for the Control group, where the Shapiro Wilk value was significant and did exceed the cut off value of *p* < 0.001. However, floor and ceiling affects were not identified and scores showed adequate spread. Inspection of box plots identified two participants as extreme outliers for Pre‐test PANAS Positive scores. Removal of these participants did not significantly change the results and were thus retained. However, the data from one participant in the Control group was excluded for the analysis of change scores because they were a multivariate outlier across all three measures. In addition, three participants did not complete the VIQ due to experimental error and were not included in the analysis for that measure. The assumption of homogeneity of variance was met using Levene's Test based on a cut off value of *p* < 0.001.

## 3 Results

### 3.1 Written Responses During the Recall and Intervention

#### 3.1.1 Recalled Performance Failures and Associated Cognitions and Affect

Participant reports of the types of performance failures are shown in Table 2. All participants were coded as having experienced a performance failure and thus met the criteria of inclusion. The most common reported performance failures were “Missed a Goal/Penalty Shot”, “Missed Catch/Throw/Pass”, and “Didn't Listen to Instruction”. Less frequently reported failures included being “Tripped Over” and “Penalty Giveaway”.

**TABLE 2:** Frequencies for the types of performance failures reported among the intervention (*n* = 18) and control (*n* = 17) groups.

Failure	Intervention	Control
Missed catch/throw/pass	4	4
Missed a goal/penalty shot	4	2
Didn't listen to instruction	1	4
Let a goal/try/punch/player through	3	2
Dropped ball while moving	1	3
Forgot the play	2	0
Build up of small mistakes	1	1
Penalty giveaway	0	1
Tripped over	2	0

The frequencies for the types of negative thoughts and feelings reported from both groups are seen in Table 3. The frequencies of reported negative thoughts exceeds the total number of participant (i.e., *N* = 35) because many participants recorded more than one negative thought. The most reported negative thought was “Embarrassed” and “Letting Others Down” for both groups. “Letting Others Down” was described as letting either the team, coach, family, or friends down.

**TABLE 3:** Frequency of types of negative thoughts and feelings reported when describing the performance failures in the intervention (*n* = 18) and control (*n* = 17) groups during the performance failure recall and in the intervention group during the writing intervention.

Negative thought	Reports during recall		Reports during writing intervention
	Intervention group	Control group	Intervention group
Embarrassed	7	3	4
Let others down	6	4	9
Disappointed	4	2	1
Anxious	4	3	2
Angry	3	3	1
Inadequate	1	3	0
Pressured	0	4	1
Inadequate	1	3	1
Underprepared	1	2	4
Annoyed	0	2	0
Demotivated	1	1	3
Regret	1	1	0
Worthless	0	1	2
Failure	0	1	0
Upset	1	0	0
Shame	1	0	2

#### 3.1.2 Negative Cognitions During the Intervention

The frequency of negative thoughts reported in the writing intervention for the Intervention group are shown in Table 3. The most reported negative thought was “Letting Others Down” and “Embarrassed”. The list of thoughts and feelings confirms that the intervention targeted a performance failure that was perceived by the participants as unpleasant or negative in nature.

### 3.2 Self‐Report Ratings at Pre‐Test and Post‐Test

**FIGURE 1:**
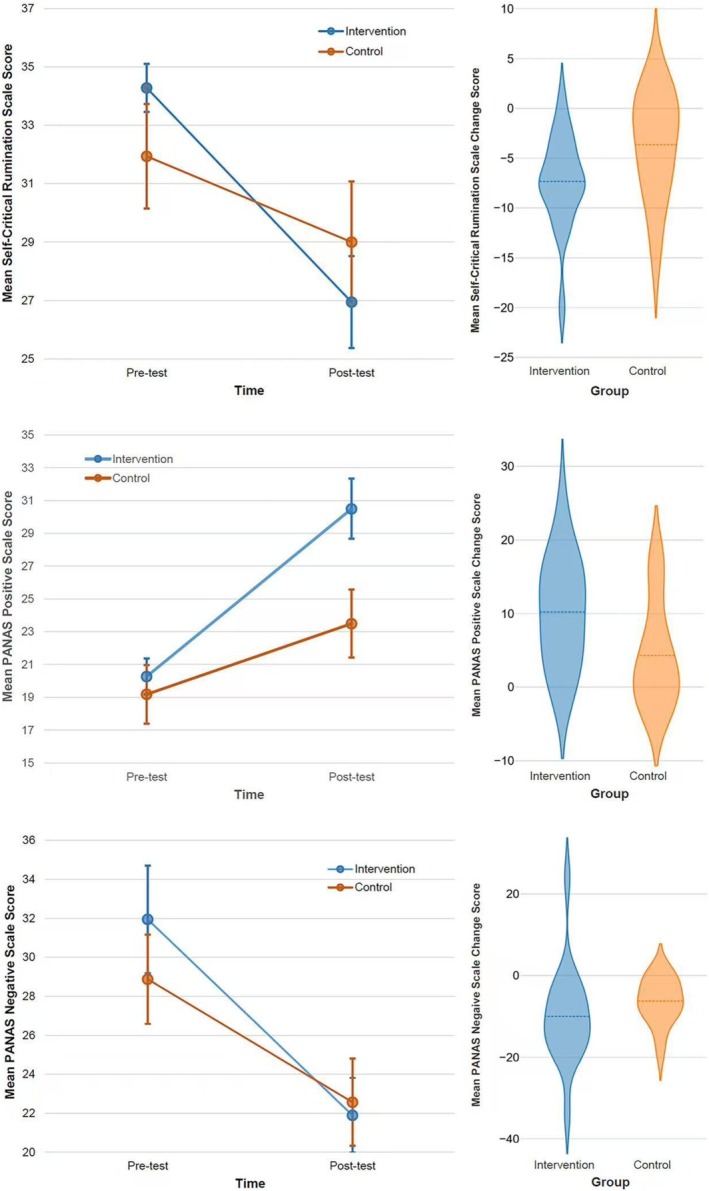
Self‐critical rumination scores (top panel), positive and negative affect schedule (PANAS) positive scale scores (middle panel), and PANAS negative scale scores (bottom panel) as a function of time and group. Mean scores are shown on the left panels and change scores are shown on the right panels.

Table 4 and Figure 1 report the descriptive statistics of the SCRS scores, PANAS Positive scores, and PANAS Negative scores at pre‐test and post‐test, and for the VIQ scores reported following the recalled performance failure. As can be seen in Table 4, the mean VIQ scores for both groups were high relative to the maximum possible score on the scale (Max = 15), indicating that all participants reported high vividness of the recalled memory. Groups did not differ in mean VIQ scores, *t*(30) = 0.023, *p =* 0.982, *d* = 0.01.

**TABLE 4:** Mean, standard deviation, 95% confidence intervals for PANAS positive, PANAS negative, SCRS, and VIQ scores for the control and intervention group.

Measure	Intervention			Control		
	*M*	SD	CI_95_	*M*	SD	CI_95_
VIQ						
Total	13.44	1.69	12.61 to 14.28	13.43	2.28	13.43–12.11
SCRS						
Pre‐test	34.23	3.50	32.56 to 36.00	32.11	6.99	28.52 to 35.70
Post‐test	26.94	6.70	23.61 to 30.27	28.47	8.33	24.18 to 32.76
Change score	−7.33	4.70	−4.99 to −9.67	−2.94	−4.49	−0.54 to −5.33
PANAS positive						
Pre‐test	20.28	4.68	17.95 to 22.60	18.89	7.29	15.14 to 22.62
Post‐test	30.50	7.78	26.60 to 34.40	24.56	11.04	18.91 to 30.26
Change score	10.02	7.32	6.58 to 13.86	4.31	7.03	0.56 to 8.06
PANAS negative						
Pre‐test	31.90	11.73	26.11 to 37.78	29.36	9.08	24.67 to 34.02
Post‐test	21.89	8.16	17.83 to 25.94	22.18	8.79	17.66 to 26.70
Change score	−10.06	11.80	−4.18 to −15.92	−6.31	5.55	−3.34 to −9.26

Abbreviations: PANAS, Positive and Negative Affect Schedule; SCRS, Self‐Critiquing Rumination Scale; VIQ, Visual Imagery Questionnaire.

#### 3.2.1 SCRS Scores

As can be seen in Figure 1 (top left panel), a disordinal interaction was found for mean SCRS scores, with both groups showing improved (reduced) scores at post‐test. There was a significant main effect of Time, *F* (1, 33) = 42.64, *p <* 0.001, *η*_*p*_^2^ = 0.56, and a significant Time × Group interaction, *F* (1, 33) = 4.81, *p =* 0.036, *η*_*p*_^2^ = 0.127. However, post hoc analyses that compared groups separately at pre‐test and post‐test failed to reach statistical significance, both *t*s < 1.79, *p* > 0.05. The main effect of Group was not significant, *F* (1, 33) = 0.02, *p =* 0.879, *η*_*p*_^2^ = 0.001. The magnitude of improvement from pre‐test to post‐test in both groups is reflected in the SCRS change scores (see Table 4 and top right panel of Figure 1). Both groups showed a significant reduction (*p* < 0.05) in SCRS scores. Moreover, the magnitude of reduction was significantly greater in the Intervention group than in the Control group, *t*(33) = 2.78, *p =* 0.009, *d* = 0.95.

#### 3.2.2 PANAS Positive Scores

Both groups improved from pre‐test to post‐test in PANAS positive scores (see middle left panel of Figure 1). Groups also appeared to differ at post‐test. A significant main effect was found for Time, *F* (1, 33) = 33.57, *p <* 0.001, *η*_*p*_^2^ = 0.50, although the main effects for Group, *F* (1, 33) = 2.48, *p =* 0.125, *η*_*p*_^2^ = 0.070, and Time × Group interaction, *F* (1, 33) = 2.70, *p* = 0.11, *η*_*p*_^2^ = 0.076, failed to reach significance. However, the change in PANAS positive scores (see Table 4 and middle right panel of Figure 1) was significant from pre‐test to post‐test (*p* < 0.05). In addition, the magnitude of the improvement in positive affect from pre‐test to post‐test was larger in the Intervention group than in the Control group, *t*(33) = 2.39, *p =* 0.023, *d* = 0.82.

#### 3.2.3 PANAS Negative Scores

As shown in Figure 1 (bottom left panel), both groups showed reduced PANAS negative affect scores at post‐test relative to pre‐test. A significant main effect was found for Time, *F* (1, 33) = 28.24, *p* < 0.001, *η*_*p*_^2^ = 0.46, but not for Group, *F* (1, 33) = 0.17, *p =* 0.683, *η*_*p*_^2^ = 0.005, or for the Time × Group interaction, *F* (1, 33) = 0.79, *p* = 0.380, *η*_*p*_^2^ = 0.001. The reduction in negative affect from pre‐test to post‐test was significant (*p* < 0.05) in both groups (see Table 4 and bottom right panel of Figure 1), although the magnitude of the change did not differ significantly between groups, *t(*33) = 1.16, *p =* 0.24, *d* = 0.40.

## 4 Discussion

The current study aimed to investigate if a brief expressive writing intervention that incorporated CBT principles is effective in mitigating self‐critiquing behavior and emotions associated with instances of recalled performance failures. Additionally, the study explored the types of performance failures reported by athletes and the negative cognitions and emotions that were associated with them. The results showed that both groups improved in self‐critical rumination, positive affect, and negative affect from pre‐test to post‐test. However, the magnitude of improvement was greater for the Intervention group than for the Control group, with this difference reaching statistical significance for self‐critical rumination and positive affect. The finding provides support for the benefits of the brief writing intervention based on the principles of reframing and cognitive restructuring for mitigating the effects of performance failures in athletes.

Many types of performance failures were recalled by the athlete sample. Those most reported were (a) missing a goal or penalty shot, (b) letting a goal, try, punch, or play through, (c) missing a catch, throw, or pass, and (d) not listening to instructions. What are the most influential failures on athletes' performance and wellbeing is less researched compared to other factors associated with performance. Many studies examine injuries in sport, and how they can have significant mental health issues on athletes (Haugen 2022). Research has also examined brief interventions when dealing with a loss in competition (Arathoon and Malouff 2004). In terms of brief approaches for performance failures, most researchers have focused on preventing failures from happening (e.g., Gómez‐López et al. 2020). The present study contributes to this literature by showing that a range of different performance failures can be associated with negative psychological impacts and that this can become evident after the failure occurs.

The athlete sample reported various negative thoughts and feelings associated with the performance failures. Supporting the findings of Rice et al. (2016), Rocha and Osório (2018), and Sagar et al. (2009) who argued that public scrutiny and high expectations were significant factors in contributing to performance anxiety among athletes, the themes of letting others down, feeling embarrassed, disappointed, and feeling under pressure were commonly reported among the participants. Notably, the high prevalence of reports of anxiety, shame, guilt, regret, and overthinking was expected due to their prevalence within the literature (Rocha and Osório 2018; Sagar et al. 2010). The role that others and their perception of athletes when an athlete is performing is not often considered. Kaye et al. (2008) and Singh and Arora (2023), however, acknowledge that athletes' self‐confidence can decrease when in the public eye. In addition, Hatzigeorgiadis and Biddle (2008) argued that the most common thoughts that arise after a performance failure are those surrounding the poor performance and what people think of them. Further research should explore the mechanisms of how those who are close to athletes, such as team members, friends, or family, influence athletes' perceptions of their performance failures.

The central finding of the present study was the benefits of the brief expressive writing task on athletes' perceptions of their performance failures. Both groups showed an improvement in the outcome measures from pre‐test to post‐test. However, the magnitude of the improvement was greater for self‐critical rumination, positive affect, and negative affect in those athletes who completed the CBT‐based expressive writing task after recalling the performance failure. Haney (2004, Kenny (2005), and Spahn (2015) argued that cognitive restructuring decreases self‐critiquing behaviors and anxiety levels, consistent with the present findings. It is noteworthy that feelings of anxiety were only reported by four participants, whereas letting others down and feeling embarrassed were more frequently reported. Nevertheless, the expressive writing task was proven to be effective in mitigating a wide range of negative psychological impacts of performance failures.

The group differences were observed in the magnitude of improvement from pre‐test to post‐test, rather than as group differences in scores when assessed at post‐test only. The lack of a difference between groups at post‐test reflects that the Control group also showed improvements in the outcome measures over time. Although demand characteristics may explain such improvements, the recruitment materials and informed consent package provided to participants were designed to minimize such effects. DiMenichi et al. (2019) argue that the effectiveness of expressive writing is dependent on how personal the failure is to the individual. By requiring the Control group to write about a past negative experience that was unique to them, this may have promoted significantly lower levels of psychological distress following the writing task. Likewise, expressive writing on an emotional topic can improve anxiety symptoms, particularly when the content of the writing includes more personal pronouns and fewer affective words (Robertson et al. 2021). Drawing upon the research by Paas and Ayres (2014) and Pennebaker and Chung (2012), the writing of the performance failure by the Control group may have freed up cognitive resources surrounding worries and allowed participants to express themselves freely. Specifically, the area of the brain that processes negative emotion and pain, the mid‐cingulate cortex (MCC), may be relieved through expressing past experiences that are seen to be painful and traumatic.

Often researchers take on the perspective of addressing the “fear of failure” rather than addressing failure as it happens. As such, the current study is distinct in attempting to target the athlete population in combating instances of recalled performance failures through using an expressive writing intervention. Moreover, this study provides a platform for future research to explore if a brief intervention can be effective if administered either during a game setting (e.g., half time break) or immediately following game completion. By being able to target negative thoughts and self‐critiquing behaviour in the moment, it may prevent these thoughts and behaviours having an immediate impact on game performance or it may prevent them becoming habitual responses to performance failures following a game. Although a writing intervention was used, there is potential for such an intervention to be administered verbally and in the moments following the performance failure by coaches, team leaders, or team captain. However, there may also be new challenges that can emerge when delivering the intervention during or immediately following a game. In such scenarios, an athlete is likely to still be experiencing heightened arousal and intense negative emotions which could reduce the effectiveness of the cognitive elements of the intervention. It may be necessary to first address the heightened emotional states before introducing the cognitive components of reframing and restructuring.

The current study contributed to the understanding of the effects of a brief CBT writing intervention in instances of performance failure among the athlete population. However, several limitations are evident. Although participants were randomly allocated to either receive the intervention or the control condition, this did not mitigate minor differences between groups at pre‐test. Conversely, significant interactions between time and group were not found in some measures despite there being differences between groups in the magnitude of change across time. Such results reduce the strength of the conclusions from the present results. Future research could use a matched pairs design to ensure greater group equivalence prior to implementing the intervention. In this way, the design may be more sensitive to detecting interactions between groups over time and differences between groups when assessed at post‐test. The nature of the sample might also be improved in future research. Although the study was adequately powered, a larger sample size should be recruited to potentially capture a wider range of performance failures experienced by athletes. In addition, sampling might selectively recruit only professional athletes or those who have competed at international levels to determine the replicability of the findings with an elite athlete group. Elite athletes will be exposed to more pressure and may have a more significant performance failure that results in more severe outcomes such as financial loss or significant career interruptions.

The nature of the intervention approach used was designed to be brief and applicable across a wide range of athletes and sport contexts. However, the majority of athletes reported playing in a team sport, whereas only six reported playing an individual sport. As such, the findings from this study may not be representative of those who play in a single player sport and might not include factors such as self‐motivation, attributing blame, support systems, and letting fellow team members down, and it is relatively unexplored within the literature if CBT could combat this among athletes. Future research could examine whether the writing intervention could be tailored to different types of athletes. Alternatively, future research should use a larger and more representative sample size that allows analyses of subgroups of athletes who play in varying levels of competition and both team and sole player sports equally.

A cross‐sectional design was used in which data was collected at one time point and relied on recalled experiences, whereby the participants relied on their memory of a performance failure rather than intervening as they happen. This could have led to participants recalling false or inflated memories or may have withheld information that was traumatic or embarrassing. Therefore, future researchers who use cross‐sectional designs may benefit from using real‐time data to ensure the failure, and the thoughts and feelings associated, are accurate. Additionally, given that instances of performance failures have been found to be associated with the development of depression and anxiety (Cowden and Worthington 2019; Li et al. 2021) future research may opt for a longitudinal design to investigate the long‐term effectiveness of the intervention approach in mitigating the risk of developing anxiety and depression. In doing so, it will be possible to give the variables temporal precedence and measure the effectiveness of the intervention at multiple time points to determine if affect and self‐critiquing behaviors change over time.

## 5 Conclusion

The immediate and long‐term impacts of performance failures in sport are often overlooked in the context of athlete mental health. Brief interventions have the potential to benefit athletes by being flexible and easily applied at any time, either close to the performance failure or following it. A brief expressive writing task that incorporates the principles of reframing and cognitive restructuring appears to be effective in mitigating the negative psychological impact of performance failures. Due to its basic principles and ease of administration, a psychologist, coach, or team leader could administer the intervention as the performance failure occurs with the aim of reducing the chance of the athlete developing anxiety, negative affect, and rumination due to the performance failure. Ultimately, the benefits of psychological approaches to address performance failures are not only for athletes mental wellbeing but also in enhancing their performance in competition.

## Conflicts of Interest

The authors declare no conflicts of interest.
